# Larvicidal and Enzymatic Inhibition Effects of *Annona Muricata* Seed Extract and Main Constituent Annonacin against *Aedes Aegypti* and *Aedes Albopictus* (Diptera: Culicidae)

**DOI:** 10.3390/ph12030112

**Published:** 2019-07-26

**Authors:** Alzeir Machado Rodrigues, Antonio Adailson Sousa Silva, Cleonilda Claita Carneiro Pinto, Dayanne Lima dos Santos, José Claudio Carneiro de Freitas, Victor Emanuel Pessoa Martins, Selene Maia de Morais

**Affiliations:** 1 Departamento de Ensino, Ciências e Formação de Professores, Instituto Federal de Educação, Ciência e Tecnologia do Pará, Avenida Almirante Barroso, 1155, Belém 66093-020, Pará, Brazil; 2 Programa de Pós-Graduação em Biotecnologia, RENORBIO, Universidade Estadual do Ceará, Avenida Doutor Silas Munguba, 1700, Fortaleza 60741-000, Ceará, Brazil; 3 Programa de Pós-Graduação em Farmacologia, Universidade Federal do Ceará, Rua Coronel Nunes de Melo, 1127, Fortaleza 60430-275, Ceará, Brazil; 4 Laboratório de Química de Produtos Naturais, Universidade Estadual do Ceará, Avenida Doutor Silas Munguba, 1700, Fortaleza 60741-000, Ceará, Brazil; 5 Vertrauen Diagnosis, Avenida Washington Soares, 655, Fortaleza 60810-000, Ceará, Brazil; 6 Instituto de Ciências Exatas e da Natureza, Universidade da Integração Internacional da Lusofonia Afro-Brasileira (Unilab), Campus das Auroras, Rua José Franco de Oliveira s/n, Redenção 62790-970, Ceará, Brazil; 7 Departamento de Química, Universidade Estadual do Ceará, Avenida Doutor Silas Munguba, 1700, Fortaleza 60741-000, Ceará, Brazil

**Keywords:** *Aedes*, larvicidal, botanicals, annonacin

## Abstract

The mosquitoes *Aedes aegypti* and *Aedes albopictus* are vectors of arboviruses that cause dengue, zika and chikungunya. Bioactive compounds from plants are environmentally sustainable alternatives to control these vectors and thus the arboviruses transmitted by them. The present study evaluated the larvicidal activity of an acetogenin-rich fraction (ACERF) and its main constituent annonacin obtained from *Annona muricata* seeds on *Ae. aegypti* and *Ae. albopictus*. The larvicidal assays were performed using different concentrations to calculate the LC_50_ and LC_90_ values observed 24 h after exposure to the treatment. Annonacin was more active against *Ae. aegypti* (LC_50_ 2.65 μg·mL^−1^) in comparison with *Ae. albopictus* (LC_50_ 8.34 μg·mL^−1^). In contrast, the acetogenin-rich fraction was more active against *Ae. albopictus* (LC_50_ 3.41 μg·mL^−1^) than *Ae. aegypti* (LC_50_ 12.41 μg·mL^−1^). ACERF and annonacin treated larvae of *Ae. aegypti* and *Ae. albopictus* showed significant differences in the inhibition of their metabolic enzymes when compared to untreated larvae. The results demonstrate the relevant larvicidal action of the acetogenin-rich fraction and annonacin showing the potential to develop new products for the control of *Ae. aegypti* and *Ae. albopictus*.

## 1. Introduction

*Aedes aegypti* Linnaeus (Diptera: Culicidae) is the main vector of arboviruses such as those that cause dengue, zika and chikungunya, diseases that affect all continents, although they are more frequent in tropical and subtropical countries [1,2]. At the same time, *Aedes albopictus* Skuse (Diptera: Culicidae), originating in Southeast Asia, is vector of diseases such as dengue, yellow fever and encephalitis, among other diseases [3]. In Brazil, its presence has been identified in almost all states [4], and it is considered a secondary vector of arboviruses in the country. Although vaccines against dengue serotypes are being developed [5], the presence of other viruses transmitted by *Aedes* spp. reinforces the need to control their vectors [6]. These mosquitoes have a broad capacity to adapt to the environment [7]. One of the most effective control strategies is the use of synthetic insecticides for the elimination of immature or adult stages (larvicides and insecticides) [8], although their frequent use has triggered the selection of resistant strains [9].

However, many plants contain bioactive compounds, which are environmentally safe for use as alternatives in the control of *Ae. aegypti* and *Ae. albopictus*, since these secondary metabolites are biodegradable [10]. This characteristic of natural products is important in the search for new environmentally safe phytopesticides for the control of immature stages of *Aedes* [11]. Thus, resistance of these disease vectors to current pesticides is a growing problem, and products from plants are eco-sustainable alternatives in this respect [12,13]. Many plant products have been evaluated against *Ae. aegypti* [14] and those from Annonaceae family show the best action [15]. The Annonaceae family is composed of a diverse group of angiosperms, including 2500 distinct species [16]. *Annona muricata* L. (Annonaceae), known as graviola or soursop, is a common edible fruit plant grown in Brazil [17]. Its fruits produce a significant number of seeds and its pulp is widely appreciated. Acetogenins (ACGs) are substances often found among the constituents of the Annonaceae family. Such compounds are characterized by the presence of an aliphatic chain with 35 to 38 carbons, attached to an α,β-unsaturated γ-lactone ring substituted with a methyl terminal, in addition to the presence of one or two tetrahydrofuran (THF) and/or tetrahydropyran (THP) rings along the hydrocarbon chain. The chain contains a specific number of oxygenated groups (hydroxyl, acetoxyl, ketone and epoxy) [18,19]. In *A. muricata*, annonacin is the acetogenin most reported to be present [18,19,20,21], being considered major component of this species.

Then, the objective of this study was to evaluate the larvicidal activity of an acetogenin-rich fraction and the main constituent, annonacin, against *Ae. aegypti* and *Ae. albopictus*, major vectors of arboviruses, as well as analyzing changes in the vital protein activity of these mosquitoes after treatment with the natural product.

## 2. Materials and Methods

### 2.1. Seed Collection and Obtaining the Ethanolic Extract of Annona muricata

*A. muricata* fruits were provided by the company Flora Boa, located in the town Limoeiro do Norte, Ceará, Brazil. The seeds (500 g) were sun dried and triturated, then macerated for 7 days with 96% ethanol. After this period, the ethanol solution was filtered and concentrated in a vacuum rotary evaporator, to obtain the ethanol extract of *A. muricata* (EESAM).

### 2.2. Obtaining the Acetogenin-Rich Fraction of A. muricata Seeds

The EESAM was subjected to filtration through silica gel column chromatography, eluted with the solvents hexane, chloroform, ethyl acetate and methanol in mixtures of increasing polarity. The fractions eluted with chloroform and ethyl acetate were pooled based on the thin-layer chromatography (TLC) analysis, using Kedde’s reagent (Solution A: 3,5-dinitrobenzoic acid in 3% methanol, and Solution B: 5.7% KOH in water, in the ratio of 1:1), to obtain an acetogenin-rich fraction (ACERF).

### 2.3. Analysis of the Acetogenin-Rich Fraction by HPLC and Separation of Its Main Constituent

The analyses were performed with Shimadzu high-performance liquid chromatograph in reverse phase, with an SCL-10Avp controller system, SPD-10Avp UV-Vis detector and LC-10Atvp gradient pump, under the following chromatographic conditions: flow rate of 1 ml per minute at a wavelength of 214 nm, mobile phase in the gradient mode of a solution composed of deionized water and methanol (15%:85%) up to 40 min, and (5%:95%) up to 60 min, oven temperature of 40 °C, 20 μL loops, and CLC-ODS M C18 column (250 mm × 4.6 mm).

To separate the constituents, 200 mg of ACERF was dissolved in 20 mL of methanol. An aliquot of 1 mL was withdrawn from that solution and dissolved in 10 mL of methanol for injection in an analytical high-performance liquid chromatograph equipped as follows: SLC-10AVP controller system, SPD-10AVP UV-Vis detector, and LC-10-ATVP isocratic pump. The column used was a Shimpack CLC-ODS M (C18), measuring 250 cm × 4.6 mm D, employing a wavelength of 214 nm, run time of 30 min, flow rate of 1 mL per minute, mobile phase composed of 87% spectroscopic methanol and 13% deionized water, injection of 20 μL, and oven temperature of 40 °C. An aliquot of the ACERF solution (12 mL) was injected and the main fraction was collected separately. This procedure was repeated several times. Then, the fractions collected were concentrated in a rotary evaporator and lyophilized to obtain annonacin, whose chemical structure was characterized by nuclear magnetic resonance spectroscopy.

The purity of the main constituent of the ACERF, identified as annonacin, was determined by injection in a liquid chromatograph in analytical mode. For this purpose, a 0.84 mg/mL solution of fraction 2 in methanol was prepared and the following system was used: Shimadzu preparative high-performance liquid chromatograph with SCL-10Avp controller system, SPD-10Avp UV-Vis detector, and LC-10Atvp gradient pump, under the following chromatographic conditions: flow rate of 1 ml per minute at a wavelength of 214 nm, mobile phase in the gradient mode of a solution composed of deionized water and methanol (15%:85%); up to 40 min and (05%:95%) up to 60 min, oven temperature of 40 °C, 20 μL loop and CLC-ODS M C18 column (250 mm × 4.6 mm).

### 2.4. Collection and Maintenance of Ae. aegypti and Ae. albopictus Mosquitoes

During the dengue control program in Fortaleza, Ceará, ovitraps are used to collect *Ae. aegypti* and *Ae. albopictus* eggs. Positive ovitraps were sent to the Entomology Laboratory (LE) of Federal University of Ceará for eggs to hatch. The larvae were obtained from a colony of successive generations maintained at the insectarium of the LE. These larvae were kept under controlled conditions of temperature (25 ± 2 °C), relative humidity (80 ± 10%) and light and dark photoperiod (12:12) in plastic containers holding 1000 mL of distilled water, and were fed with fish meal until reaching the third and fouth instars.

### 2.5. Larvicidal Assay

The larvicidal tests were performed at concentrations of 0.5, 1, 5, 10, 20, 25, 50 and 75 μg·mL^−1^ of ACERF and annonacin from *A. muricata*, all of which were performed in triplicate. The ACERF and annonacin at different concentrations were diluted in a solution of 5% dimethyl sulfoxide (DMSO) and 95% distilled water, and a negative control was also tested with DMSO and water. Twenty larvae were included in each test and mortality was verified after 24 h of exposure [22].

### 2.6. Enzymatic Effect of the Acetogenin-Rich Fraction and Annonacin of A. muricata on the Mosquito Larvae

The enzymatic assays were performed using a Bioplus Bio-200 biochemical analyzer. The action of ACERF and annonacin on the larvae of *Ae. aegypti* and *Ae. albopictus* exposed at a concentration of 25 μg·mL^−1^ during 24 h was verified from the analysis of the main enzymes of the metabolism of Culicidae larvae by adaptation of the method of Suryawanshi et al. [23]. The enzymes assayed were alkaline phosphatase, acid phosphatase, proteases, esterases and amylases. All experiments were performed with larval stages.

### 2.7. Statistical Analysis

The LC_50_ values (concentration that kills 50% of larvae and pupae) and LC_90_ (concentration that kills 90% of larvae) were obtained from probit analysis using the SPSS statistical software. Analysis of variance (ANOVA) was used to investigate the existence of significant differences in the parameters of the enzymatic activities of treated and untreated mosquito larvae after ACERF and annonacin application (*p* < 0.05). The normality criterion was applied to the variables and the Tukey test was used to verify where the differences occurred in the studied groups.

## 3. Results and Discussion

### 3.1. Analysis of the Constituents of the Acetogenin-Rich Fraction (ACERF)

**Figure 1:**
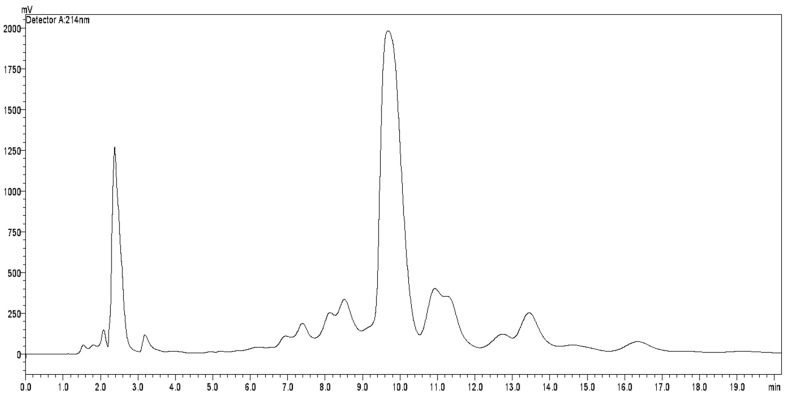
Chromatogram of the ACERF.

**Figure 2:**
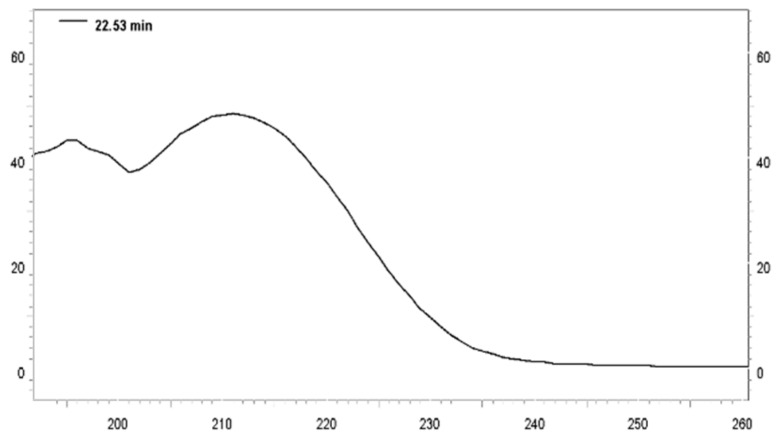
UV curve of the main acetogenin isolated from the acetogenin-rich extract of *A. muricata*.

**Figure 3:**
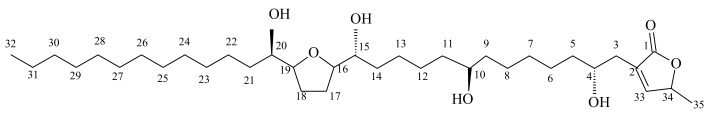
Representation of chemical structure of annonacin.

In the TLC analysis of the various ACERF fractions obtained in the chromatographic column (those that revealed a characteristic pink color with Kedde’s reagent) were collected and the chromatogram of HPLC analysis (Figure 1) presented several peaks with similar UV spectra (Figure 2). The ACERF was subjected to semi-preparative HPLC fractionation to separate the main acetogenin constituent with a retention time of 9.6 min. The major substance was separated and analyzed by nuclear magnetic resonance, and was revealed to be annonacin (Figure 3) by comparison with spectral data reported in the literature for this substance [24].

### 3.2. Efficacy of the ACERF and Annonacin of A. muricata Seeds on Ae. aegypti and Ae. albopictus

The larvicidal action of the acetogenin-rich fraction of *A. muricata* seeds is shown in Table 1. The LC_50_ of ACERF for *Ae. albopictus* was 3.41 μg·mL^−1^—the best result in relation to other strains. The *Ae. aegypti* (Rockefeller strain) showed highest resistance to treatment with ACERF of *A. muricata*, with LC_50_ and LC_90_ values, respectively, of 26.75 and 54.46 μg·mL^−1^, as determined by the probit analysis. Concentrations higher than 50 μg·mL^−1^ were responsible for 100% mortality of *Ae. albopictus* and concentrations higher than 75 μg·mL^−1^ were totally lethal to the other strains.

A study of the leaf extract from other Annonaceae plants, such as *A. reticulata*, reported effective action against *Ae. aegypti* larvae, with LC_50_ and LC_90_ values of 95.24 and 262.64 µg·mL^−1^, respectively [25]. The hexane extract of the leaves of *A. muricata* gathered in Tarlac City (Philippines) showed the highest toxicity, with LC_50_ value of 25.99 μg·mL^−1^and LC_90_ value of 49.47 μg·mL^−1^, while the ethanol extract yielded LC_50_ of 46.36 µg·mL^−1^ and LC_90_ of 195.39 µg·mL^−1^. Characterization of the ethanol extract showed the presence of flavonoids (leucoanthocyanidins), condensed tannins, unsaturated steroids, triterpenoids, and fats and oils.

Regarding the strain of *Ae. albopictus*, Kempraj and Bhat [26] reported the larvicidal action of the acetone soluble fraction of the ethanolic extract of *A. squamosa* seeds, with respective LC_50_ and LC_90_ values of 5.26 and 38.37 μg·mL^−1^, on fourth instar larvae. The authors also performed tests of ovicidal and adulticide activity, as well as tests to verify the effect of the *A. squamosa* extracts on oviposition of females, with toxicity observed in all trials.

The plants of the Annonaceae family show high toxicity to *Aedes* spp. larvae. The larvicidal activities against *Ae. aegypti* have been determined in the ethanolic extracts obtained from 51 Brazilian medicinal plants. Eleven of the 84 extracts studied showed significant (LC_50_ < 100 μg·mL^−1^) activities against larvae, with extracts from *A. crassiflora* (root bark, LC_50_ = 0.71 μg·mL^−1^; root wood, LC_50_ = 8.94 µg·mL^−1^ and *A. glabra* seed, LC_50_ = 0.06 μg·mL^−1^, showing the highest activities [27].

Natural products from plants of other families (Myrtaceae, Rutaceae, Euphorbiaceae, Piperaceae, Asteraceae and Liliaceae) in general are less active. The essential oils of *Eucalyptus cinerea* had LC_50_ of 380 μg·mL^−1^ [28], *Citrus sinensis* had LC_50_ of 446.84 µg·mL^−1^ [29] and *Piper gaudichaudianum* had LC_50_ of 121 µg·mL^−1^ [30]. Various solvent extracts of stems, roots and leaves of *Parthenium hysterophorus* showed low efficacy, with LC_50_ above 400 μg·mL^−1^ [31], and *Aloe vera*, with LC_50_ of 300.06 µg·mL^−1^ [32]. The LC_50_ values of petroleum ether extracts of *Jatropha curcas*, *Piper tithymaloides*, *Phylantus amarus*, *Euphorbia hirta*, and *E. tirucalli* were 8.79, 55.26, 90.92, 272.36, and 4.25 μg·mL^−1^, respectively, against *Ae. aegypti* and 11.34, 76.61, 113.40, 424.94, and 5.52 μg·mL^−1^, respectively, against *C quinquefasciatus* [33].

Annonacin is considered an environmental neurotoxin and is found in the pulp of several fruits of the Annonaceae family, whose consumption has been linked to the occurrence of sporadic atypical Parkinsonism with dementia. A method for its quantification in rat brain homogenates by UPLC-MS/MS was developed and validated. This method was applied to the quantitation of annonacin in rat brains after intravenous (0.5 mg/kg) and oral (10 mg/kg, 100 mg/kg) administration. Nevertheless, annonacin appeared to have very low distribution in rat brains by both routes [34].

Annonacin was shown to be significantly active against *Ae. aegypti* (Rockefeller strain), presenting lower lethal concentrations than those observed for the acetogenin-rich fraction (LC_50_ 2.65 μg·mL^−1^; LC_90_ 4.83 µg·mL^−1^). The larvae of *Ae. albopictus* presented LC_50_ of 8.34 μg·mL^−1^ and LC_90_ of 16.30 μg·mL^−1^ (Table 2).

According to the literature, the larvicidal action against mosquitoes of Annonaceae plant extracts is comparable to that reported for pure acetogenins [27]. The ethanolic extract of *A. glabra* had LC_50_ of 27 μg·mL^−1^ against *Ae. aegypti* larvae [35], in contrast to isolated acetogenins such as goniothalamin, gigantriocin and longimicin, with LC_50_ values of 13.3 μg·mL^−1^, 18.5 μg·mL^−1^ and 27.3 μg·mL^−1^, respectively [36].

The high activity of ACERF and annonacin against larvae of *Ae. aegypti* and *Ae. albopictus* enables the use of low amounts. The small distribution of this substance in the rat brain, conferring low toxicity, justifies the use in phytotherapic preparations as larvicidal agents.

### 3.3. Changes in the Enzymatic Level of Ae. aegypti and Ae. albopictus Treated with ACERF and Annonacin

The mechanism of action of natural products on *Aedes* spp. larvae has been investigated in recent studies [1,37]. Among the explanations of this mechanism are the ultrastructural alterations of organs such as anal papillae [38], intestinal and tracheal system damage [1], inhibition of enzymatic activity [39], and morphological aberrations [40]. In this study, we investigated the effect of ACERF and annonacin on the inhibition of proteins important for the development of *Aedes* spp. larvae.

**Figure 4:**
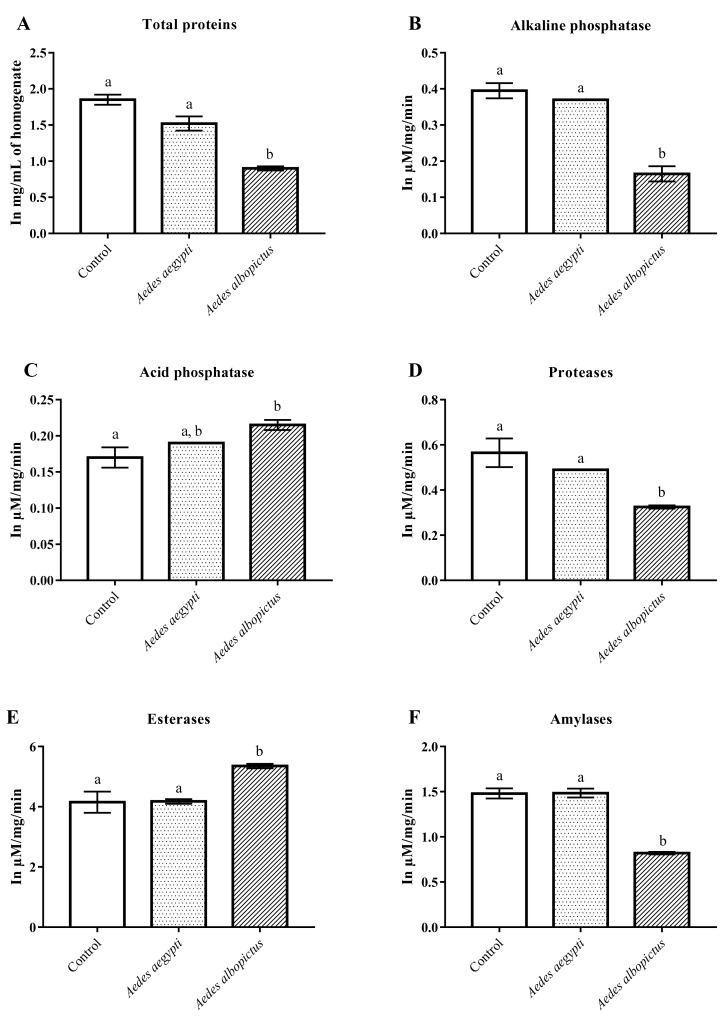
Enzymatic changes of total proteins (**A**), alkaline phosphatase (**B**), acid phosphatase (**C**), proteases (**D**), esterases (**E**) and amylases (**F**) between larvae treated and untreated with the ACERF of *A. muricata*. Different lowercase letters on the bar denote significant difference (*p* < 0.05). Bar represent the standard deviations (n = 2). Values estimated by one-way ANOVA followed by Tukey’s test. Control: untreated larvae.

The one-way ANOVA showed that ACERF has an effect on the total proteins between the groups (*p* < 0.05). The Tukey test showed the yield of total proteins of *Ae. albopictus* treated with ACERF was significantly different from the control and *Ae. aegypti*. However, there was no difference between the control group and the treated *Ae. aegypti* (Figure 4A).

Alkaline phosphatase activity was altered by ACERF treatment (*p* < 0.05). The Tukey test indicated no difference between the control group and *Ae. aegypti* strain, however, both groups were significantly different from *Ae. albopictus* (Figure 4B). Similarly, there was a change in the enzymatic effect of acid phosphatase (*p* < 0.05), but it was the only significant difference between *Ae. albopictus* and control (Figure 4C).

Among the proteases, there was a difference between the groups (*p* < 0.05). This difference, when verified by the post-hoc Tukey test, was significant between *Ae. albopictus* and the control and *Ae. aegypti* (Figure 4D). Differences were also observed between groups in relation to esterase activity (*p* < 0.05), with a significant increase in the activity of these enzymes in strain *Ae. albopictus* compared to the other enzymes—which were similar to each other—as verified by the Tukey test (Figure 4E).

The ACERF also elicited significant changes between groups with respect to amylase activity (*p* < 0.05), and the larval strain *Ae. albopictus* showed a significant reduction compared to the control and to *Ae. aegypti* (Figure 4F).

The consistent changes in the enzymatic activity of *Ae. albopictus* (LC_50_ = 3. 41 μg·mL^−1^) treated with ACERF corroborate the greater larvicidal effect of this fraction in this species when compared to *Ae. aegypti* (LC_50_ = 12.41 μg·mL^−1^).

**Figure 5:**
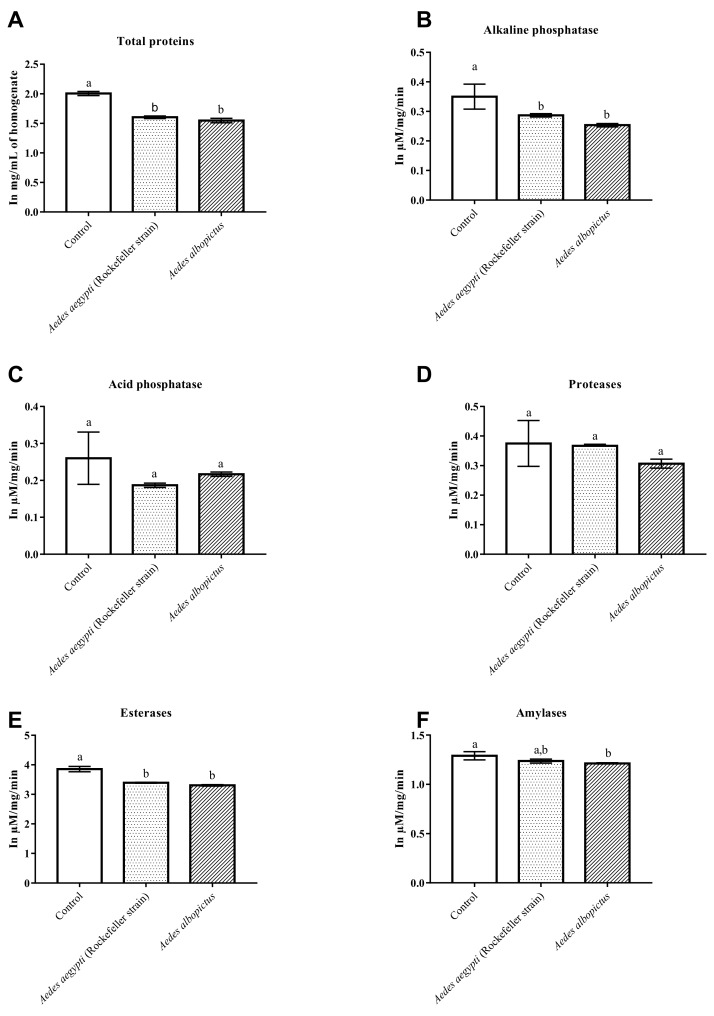
Enzymatic changes of total proteins (**A**), alkaline phosphatase (**B**), acid phosphatase (**C**), proteases (**D**), esterases (**E**) and amylases (**F**) between larvae treated and untreated with the annonacin of *A. muricata*. Different lowercase letters on the bar denote significant difference (*p* < 0.05). Bar represent the standard deviations (n = 2). Values estimated by one-way ANOVA followed by Tukey’s test. Control: untreated larvae.

In relation to the treatment of *Aedes* larvae with annonacin, one-way ANOVA showed a significant difference between groups in activity of total protein (*p* < 0.05), alkaline phosphatase (*p* < 0.05) and esterase (*p* < 0.05). The Tukey post-hoc test confirmed that the activities of the total proteins, alkaline phosphatase and esterase of the control group were significantly different against *Ae. aegypti* (Rockefeller strain) and *Ae. albopictus* (Figure 5A,B,E).

There was no significant difference in the activity of acid phosphatase (Figure 5C) (*p* > 0.05) and protease (Figure 5D) (*p* > 0.05) between the control and treated groups. The amylase activity showed significant differences between the groups (*p* < 0.05). While the Tukey post-hoc confirmed significant differences only between the control and *Ae. albopictus* groups, the *Ae. aegypti* (Rockefeller strain) group did not present significant differences between these two groups (Figure 5F).

Similarly, studies of enzymatic changes in larvae and pupae of *Ae. aegypti* treated with the aqueous extract of *Sapindus emarginatus* Vahl. (Sapindaceae) showed that the activity of esterases and acid phosphatase were affected, and more noticeably in larvae than pupae [41].

Another study correlated the inhibitory capacity of a protease inhibitor from garlic (*Allium sativum*) with its larvicidal activity [42]. These authors considered that the reduction of 50% in the presence of digestive proteases by the *A. sativum* protease has biotechnological potential for the control of *Ae. aegypti*. In this respect, ACERF of *A. muricata* caused a reduction in proteases of both treated strains and contributed to the inactivation of those proteins, which is detrimental for the survival of *Aedes* spp.

The larvicidal effect of ACERF was greater on larvae of *Ae. albopictus* than on other treated strains, whereas annonacin had a larvicidal activity greater in *Ae. aegypti* (Rockefeller strain). Regarding the inhibitory effect on the enzymes, differences were observed between ACERF and annonacin on total proteins, acid phosphatase and alkaline phosphatase in strains with greater susceptibility to natural products. These differences most likely result from the fact that in ACERF there are other acetogenins besides its major constituent—annonacin. These acetogenins are substances that act synergistically in the enzymatic inhibitory effect and in the larvicidal action, whereas in the isolated annonacin, we have the presence of a single substance that conditions specific effects on the larvae and their enzymes.

## 4. Conclusions

*Ae. aegypti* and *Ae. albopictus* exhibit different susceptibilities to the fraction rich in acetogenins and annonacin—the main constituent. In addition, ACERF-treated larvae of *Ae. albopictus* showed a significant difference in the activity of all the enzymes studied compared to control larvae, a fact that corroborates the relevant LC_50_ observed in the larvicidal tests of this lineage of the natural product in question. Regarding the treatment with annonacin, there were significant differences between treated and untreated larvae, however only for the activity of total proteins, alkaline phosphatase, esterase and amylase. This study indicates the promising potential of *A. muricata* as an alternative source of sustainable, biodegradable and environmentally safe natural product for the control of the dengue vectors *Ae. aegypti* and *Ae. albopictus*.

## Figures and Tables

**Table 1:** Lethal concentration of the fraction rich in acetogenins of *A. muricata* against fourth instar larvae of *Ae. aegypti* and *Ae. Albopictus*.

Mosquito Strains	LC_50_ (CL at 95%)	LC_90_ (CL at 95%)
*Ae. aegypti*	12.41 (9.85–14.86)	30.21 (26.17–36.52)
*Ae. albopictus*	3.41 (2.83–4.00)	6.17 (5.37–7.43)
*Ae. aegypti* (Rockefeller strain)	26.75 (21.36–34.00)	54.46 (44.78–71.01)

Control: no mortality. As the level of significance is greater than 0.05, no heterogeneity factor was used in the calculation of confidence limits. LC_50_: concentration that kills 50% of the exposed larvae (in μg·mL^−1^) with confidence limit at 95%. LC_90_: concentration that kills 90% of the exposed larvae (in μg·mL^−1^) with confidence limit at 95%.

**Table 2:** Lethal concentration of annonacin from *A. muricata* against fourth instar larvae of *Aedes* spp.

Mosquito Strains	LC_50_ (CL at 95%)	LC_90_ (CL at 95%)
*Ae. aegypti* (Rockefeller strain)	2.65 (1.87–3.64)	4.83 (3.80–7.35)
*Ae. albopictus*	8.34 (7.10–10.26)	16.30 (13.56–21.13)

Control: no mortality. As the level of significance is greater than 0.05, no heterogeneity factor was used in the calculation of confidence limits. LC_50_: concentration that kills 50% of the exposed larvae (in μg·mL^−1^) with confidence limit at 95%. LC_90_: concentration that kills 90% of the exposed larvae (in μg·mL^−1^) with confidence limit at 95%.
